# Upregulated *PPARG2* facilitates interaction with demethylated *AKAP12* gene promoter and suppresses proliferation in prostate cancer

**DOI:** 10.1038/s41419-021-03820-7

**Published:** 2021-05-22

**Authors:** Feng Li, Tingting Lu, Dongmei Liu, Chong Zhang, Yonghui Zhang, Fulu Dong

**Affiliations:** 1 Department of Pathology, Key Laboratory of Microenvironment and Translational Cancer Research, Medical School of Nantong University, Nantong, 226001 Jiangsu China; 2 Department of Laboratory Medicine, Affiliated Hospital of Nantong University, Nantong, 226001 Jiangsu China; 3 Basic Medical Research Centre in Medical College of Nantong University, Nantong, 226001 Jiangsu China

**Keywords:** Prostate cancer, Prostate cancer

## Abstract

Prostate cancer (PCA) is one of the most common male genitourinary tumors. However, the molecular mechanisms involved in the occurrence and progression of PCA have not been fully clarified. The present study aimed to investigate the biological function and molecular mechanism of the nuclear receptor peroxisome proliferator-activated receptor gamma 2 (*PPARG2*) in PCA. Our results revealed that *PPARG2* was downregulated in PCA, and overexpression of PPARG2 inhibited cell migration, colony formation, invasion and induced cell cycle arrest of PCA cells in vitro. In addition, *PPARG2* overexpression modulated the activation of the Akt signaling pathway, as well as inhibited tumor growth in vivo. Moreover, mechanistic analysis revealed that *PPARG2* overexpression induced increased expression level of miR-200b-3p, which targeted 3′ UTR of the downstream targets DNMT3A/3B, and facilitated interaction with demethylated *AKAP12* gene promoter and suppressed cell proliferation in PCA. Our findings provided the first evidence for a novel PPARG2-AKAP12 axis mediated epigenetic regulatory network. The study identified a molecular mechanism involving an epigenetic modification that could be possibly targeted as an antitumoral strategy against prostate cancer.

## Introduction

Prostate cancer (PCA) is one of the most common male genitourinary tumors with its high fatality rate in the western world^1^. Reports revealed that the United States is expected to spend more than US$8 billion annually on the screening and treatment of PCA^2^. Incidence of PCA in China is lower than in the United States; however, in the past two decades, numbers of PCA patients have increased significantly due to environmental pollution, westernized change in diet, and aging of the population^3^. The prostate-specific antigen (PSA) is currently recognized as a useful tool for early screening of PCA^4^. However, screening based on serum PSA is still largely debated^5^, and up to 22% of newly diagnosed patients with PCA are advanced or metastatic ones^6^. Once diagnosed, it is usually treated by active surveillance, prostatectomy, radiation therapy, hormone therapy, or chemotherapy^7^. So far, the complex molecular mechanisms involved in the occurrence and progression of PCA have not been fully clarified. We believe it is necessary to explore the pathological mechanism and look for new molecular therapeutic targets of PCA.

The nuclear receptor peroxisome proliferator-activated receptor-γ (PPARG) is a ligand‑dependent transcription factor (TF) that plays a vital role in regulating the differentiation of adipocytes and the transcription of multiple genes^8–11^. The human PPARG gene was found to be located on the short arm of chromosome 3 (3p25) in 1995. PPARG exists in two protein isoforms, PPARG1 and PPARG2^11^. Compared to PPARG1, PPARG2 contains 30 additional amino acids at the N terminus and the ligand-independent activation activity is 5–10 times than that of PPARG1^12^. It has been reported that PPARG plays a role in a variety of chronic diseases including tumors^3,13^, diabetes^14^, inflammation^15^, atherosclerosis^16^, and so on. As far as PPARG2 is concerned, its role in PCA has not been clarified.

DNA methylation is a common type of epigenetic modification. The existence of CpG islands in human genome is always closely related to the methylation status and it is also related to a majority of the coding genes in the human genome^17^. DNA methylation plays an important role in the of gene expression, cell proliferation, differentiation, and development, and is also closely related to human development and tumors^18–20^. When one gene promoter region is methylated, its transcription is often inactivated, whereas demethylation is usually manifested as a transcriptional activation.

In the present study, we showed that the downregulation of *PPARG2* expression in PCA acted as a tumor suppressor in suppressing malignancy of PCA cells in vitro and in vivo. Moreover, mechanistic analysis revealed that upregulated *PPARG2* facilitated interaction with demethylated A-Kinase anchoring protein 12 (*AKAP12*) gene promoter and suppressed cell proliferation in PCA. Our present results provide the first evidence for a novel PPARG2-AKAP12 axis-mediated epigenetic regulatory network. The study identified a molecular mechanism involving an epigenetic modification that could be possibly targeted as an antitumoral strategy against PCA.

## Materials and methods

### Cell lines and culture

The human PCA cell lines (LNCap, PC3, and DU145) were purchased from the Chinese Academy of Sciences (Shanghai, China) and were cultured with Roswell Park Memorial Institute 1640 (10-040-CV, Corning, USA) containing 10% fetal bovine serum (FBS, Gibco, Billings, MT). The non-malignant immortalized human prostate epithelial cell line named NHPrE1 has been described previously^21,22^. The cell line was routinely passaged in 50/50 Dulbecco’s modified Eagle’s medium (DMEM)/F12 (Gibco) culture medium containing 5% FBS, 1% insulin-transferrin-selenium-X (Gibco), 0.4% bovine pituitary extract (Hammond Cell Tech, Windsor, CA), and 10 ng/ml epidermal growth factor (Sigma, Woodstock, VA) with 1% AB/AM (Gibco). The miR-200b-3p mimic and inhibitor were purchased from GenePharma Co., Ltd (Shanghai, China). HEK 293T cell line was cultured with DMEM containing 10% FBS. Cells were cultured on different sizes of cell culture dishes in a humidified atmosphere containing 5% CO_2_ at 37 °C. All cells were authenticated by short tandem repeat profiling.

### Tissue samples

Eight human PCA samples and eight benign prostatic hyperplasia tissues were obtained from the Urology Department of Affiliated Hospital of Nantong University, between 2017 and 2018. The samples were frozen quickly in liquid nitrogen and were stored at −80 °C. The study was in accordance with the International Ethical Guidelines for Biomedical Research Involving Human Subjects. The protocol was approved by the Ethics Committee of the Affiliated Hospital of Nantong University. All subjects obtained informed consent to participate in this study.

### Construction of lentivirus vectors

The PPARG2 overexpression lentivirus vector-GV358 containing the human PPARG2 wild-type full-length sequence (PPARG2) for gain-of-function and the lentivirus empty vector (EV) as a control were constructed by GeneChem Co., Ltd (Shanghai, China). In brief, the successful construction of the plasmid was first verified by restriction enzyme digestion, PCR identification, and sequencing. Then, the constructed plasmid and lentivirus packaging plasmids phelper 1.0 and phelper 2.0 were co-transfected into the cultured HEK 293T cells. Lentiviral particles were obtained by collecting supernatant using the kit for ultracentrifugation concentration and purification of lentiviral particles, and combined with fluorescent titer assay and enzyme-linked immunosorbent assay. Virus titer was determined as 1 × 10^8^ transducing U ml^−1^.

### Gene expression profiling and miRNA-seq analysis

The gene expression profiles of PC3-PPARG2 cDNA (PPARG2) and PC3-EV were compared using Agilent SurePrint G3 Human Gene Expression 8 × 60K Microarray (Agilent Technologies, Santa Clara, CA) (Gene Expression Omnibus database accession number GSE108309). High-throughput miRNA sequencing (miRNA-seq) between PPARG2 and EV group was conducted using the single-ended 50 bp sequencing mode of the Illumina Hiseq3000 sequencing platform (Genergy Bio-technology, Shanghai, China). The Sequence Read Archive (SRA) accession number was PRJNA719139. Differential expression genes (DEGs) between PPARG2 and EV were screened, based on a *t*-test of linear models for microarray analysis package in R (Version 3.3, http://www.bioconductor.org)^23^. DEGs fold change of gene expression was calculated with a threshold of fold change > 1 and *P*-value < 0.05 for DEG selection.

### GO and KEGG pathway enrichment

The Database for Annotation, Visualization, and Integrated Discovery (DAVID, Version 6.8, http://david.abcc.ncifcrf.gov/) can provide a comprehensive set of functional annotation tools for investigators to understand biological meaning behind a large list of genes^24^. Gene Ontology (GO) and Kyoto Encyclopedia of Genes and Genomes (KEGG) pathway enrichment analysis were performed using DAVID online tool, to analyze the DEGs at the functional level. *P* < 0.05 was considered statistically significant.

### Real-time quantitative PCR

Real-time quantitative PCR (qPCR) was used to detect the expression levels of mRNAs and miRNAs that were involved in this study. Total RNA was extracted using Trizol reagent (Invitrogen) according to the manufacturer’s protocol. The primer sequences (*AKAP12*, *PPARG2*, miR-200b-3p, U6, and *GAPDH*) were listed in Supplementary Table S1. The conditions of qPCR amplification were as follows: the holding stage keeps 95 °C for 5 min (1 cycle), the cycling stage holds 95 °C for 15 s and 60 °C for 45 s (40 cycles), the melt curve stage keeps 95 °C for 15 s, 60 °C for 1 min, and 95 °C for 15 s (1 cycle). The whole amplifications were on Step OnePlus Real-Time PCR Systems (ABI, Life Technologies Corporation, USA). The 2^−ΔΔCt^ method was applied to calculate the relative quantities of the target RNAs^25^.

### Western blotting

Total proteins of the tissues and cells were extracted using a protein extraction kit (Beyotime Biotechnology, Shanghai, China) according to the manufacturer’s instructions. The extracted protein concentrations were detected using a bicinchoninic acid kit (Sigma-Aldrich, St. Louis, USA). The protein samples (each 40 μg) were separated using polyacrylamide gel electrophoresis (10% concentration) and transferred to a polyvinylidene fluoride membrane. The membrane was then blocked with 5% fat-free milk at room temperature for 1 h and incubated with primary antibody at 4 °C overnight. The next day, the membrane was washed with Tris-buffered Saline Tween 20 (TBST) for four times (3 min/time) and then incubated with the corresponding secondary antibody (1 : 2000; catalog number 7056 or 7054, Cell Signaling Technology, Inc.) for 1 h at room temperature. Again, the membrane was washed with TBST for four times. The target bands were scanned and visualized using chemiluminescence method with Bio-Rad Gel Doc EZ imager (Life Science Research, CA, USA). Image J software (National Institutes of Health, MD) was applied to analyze the intensity of the target bands. The primary antibodies used were as follows: PPARG2 (1 : 800; catalog number sc-166731, Santa Cruz, CA), cyclinD1 (1 : 1000; catalog number sc-166731, Santa Cruz, CA), cyclinB1 (1 : 1000; catalog number 12231, Cell Signaling Technology, Inc.), p21^Cip1^ (1 : 1000; catalog number 2947, Cell Signaling Technology, Inc.), p27^Kip1^ (1 : 1000; catalog number 3686, Cell Signaling Technology, Inc.), Bcl-2 (1 : 1000; catalog number 4223, Cell Signaling Technology, Inc.), AKT (1 : 1000; catalog number SAB4500797, Sigma-Aldrich, Saint Louis, MO, USA), p-AKT (1 : 1000; catalog number 05–802 R, Sigma-Aldrich, Saint Louis, MO, USA), DNA methyltransferase 3A (DNMT3A (1 : 1000; catalog number 32578, Cell Signaling Technology, Inc.), and DNMT3B (1 : 1000; catalog number 72335, Cell Signaling Technology, Inc.). β-Actin (1 : 1000; catalog number 3700, Cell Signaling Technology, Inc.) was used as an internal reference.

### Cell proliferation, colony formation, migration, and invasion

For cell proliferation, cells (3 × 10^3^ per well) were seeded to a 96-well plate and cultured for 0, 24, 48, 72, and 96 h. Then, 100 μl of medium containing 10 μl Cell Counting Kit-8 (CCK-8) reagent (Beyotime Biotechnology, Shanghai, China) was added to each well for incubation of another 2 h at 37 °C. The absorbance was then measured at 450 nm according to the manufacturer’s instruction.

For 5-ethynyl-2′-deoxyuridine (EdU) incorporation assay, cells (5 × 10^4^ per well) were cultured in 96-well plates at 37 °C for 48 h. Then, 100 μl of medium containing 50 μM EDU (catalog number C10310-1, RiboBio Biotechnology, Guangzhou, China) was added to each well for another 2 h at 37 °C and fixed with 4% paraformaldehyde for 30 min. Then, the fixed cells were permeabilized with 0.5% Triton X-100 for 10 min. Finally, the cells were stained with Apollo® 567 and Hoechst33342, respectively.

For colony formation, cells were seeded to a six-well plate and cultured for 2 weeks. Then the cells were fixed with 4% paraformaldehyde for half an hour and stained with crystal violet (Sigma-C3886) for 10 min, and then the colonies comprising over 50 cells were counted.

For migration of wound-healing assay, in brief, the cell monolayers of each well in a six-well plate were scratched using a 100 µl pipette tip and photographed at 0 and 24 h, respectively.

For cell invasion assay, the diluted Matrigel (catalog number 356234; BD Biosciences) was added to each Transwell upper chamber. Then, cells (1 × 10^5^) that cultured in serum-free medium were added to the upper chambers and the complete medium was added to the lower chambers. After 36 h, the cells were fixed with 4% paraformaldehyde for half an hour and stained with 0.5% crystal violet for 10 min at room temperature. Cells were counted under a microscope (Leica DM2500, Leica Microsystems, Inc.) at ×200 magnification.

### Flow cytometry

Cells (1 × 10^6^ per well) were seeded to a six-well plate. After 24 h, cells were trypsinized and washed with precooled phosphate-buffered saline (PBS) and fixed with 70% ethyl alcohol at 4 °C overnight. Then, the cell suspension was incubated with propidium iodide (0.5 mg/ml) (Beyotime Biotech, Shanghai, China) for 15 min. DNA content was analyzed using a flow cytometer (BD Biosciences, San Jose, CA).

### Tumor xenograft models

The experiments were approved by the Research Ethics Committee of Nantong University according to Council on Animal Care Guidelines of Nantong University. A total of 12 BALB/c 5-week-old male nude mice were randomly divided into EV and PPARG2 groups (6 per group). EV or PPARG2-transfected PC3 cells were injected subcutaneously into the flanks of the mice (1 × 10^6^ cells/100 μL per flank). Tumor growth of mice was observed every 7 days, using a caliper for the tumor volumes. Thirty-five days later, all mice were killed and the tumors were weighted and photographed. Then, tumor tissues were used for hematoxylin and eosin (H&E) staining and immunohistochemical analysis of Ki67 protein expression. The tumor volumes were measured and calculated using the following formula: volume (*V*) = width (*W*)^2^ × length (*L*)/2.

### Methylation-specific and bisulfite-sequencing PCR

For methylation-specific PCR (MSP), the methylation status of CpG islands in *AKAP12* gene promoter region were screened using MSP method initially in PCA cells. In brief, DNA samples that modified with bisulfite were extracted according to instructions of the manufacturer (Zymo Research, Orange, CA) first. Then, a total of 40 ng of bisulfite-modified DNA was used for PCR amplification. After that, 10 µl of the amplificated product was taken to analyze using Agarose gel electrophoresis. For interpretation of the MSP results, the methylated and unmethylated state is represented by the methylation (M) and unmethylation (U) band, respectively. Occasionally, if the site is methylated partially, the two bands may appear.

Bisulfite-sequencing PCR (BSP) is a sequencing method to detect the methylation status of CpG islands. Briefly, DNA samples were treated with bisulfite and amplified by PCR. Then, the PCR products were purified using a TIANgel Midi Purification Kit (Tiangen Biotech, Beijing, China). After that, the purified products were cloned into a pGEM-T easy vector (Promega, Madison, WI, USA). Nine colonies were randomly chosen for plasmid DNA extraction using a Promega Spin Mini kit (Promega) and then sequenced by an ABI 3130 Genetic Analyzer (Applied Biosystems, Foster City, CA, USA).

### Dual luciferase reporter

In brief, the 3′-untranslated region (3′-UTR) of DNMT3A/3B (wild type and mutant) were amplified first and cloned into the pmiR-RB-Report^TM^ vector (Ribobio, Guangzhou, China), respectively. Then HEK 293T cells were co-transfected with pmiR-RB-Report^TM^-WT- DNMT3A/3B or pmiR-RB-Report^TM^-MUT-DNMT3A/3B and miR-200b-3p mimic/mimic control. After 48 h, the Dual Luciferase Reporter Detection System (Promega, Madison, WI, USA) was used to detect luciferase activity. Firefly luciferase (*hLuc*+) was the reporter gene and *Renilla* luciferase (*hRluc*) was the internal reference gene. The relative activity changes of *hLuc*+/*hRluc* were detected to determine whether miRNAs could target 3′-UTR of the corresponding gene.

For *AKAP12* gene promoter analysis, pGL3-Basic vector was selected for construction. Relative luciferase activity was detected by the Dual Luciferase Assay system (Promega). The phRL-TK vectors (Promega) were used as the internal reference.

### Chromatin immunoprecipitation

Chromatin immunoprecipitation (ChIP) assay was performed using ChIP Enzymatic Chromatin IP Kit (Magnetic beads, Cell Signaling, Danvers, MA) according to the manufacturer’s instructions. Briefly, the cells were crosslinked with formaldehyde of 1% final concentration first. Then, they were washed with pre-cold PBS and collected, followed by sonication crush. The solution complexes were immunoprecipitated using the anti-PPARG2 antibody (1 : 100; catalog number sc-166731, Santa Cruz, CA) or rabbit immunoglobulin G (IgG, negative control). After that, the immunoprecipitated complexes were collected using protein G-agarose beads. The precipitates were eluted from the beads and the DNA–protein complexes were de-crosslinked at last. The DNA samples were recollected and used for PCR analysis. The PCR conditions were as follows: the holding stage keeps 95 °C for 5 min (1 cycle), the cycling stage holds 95 °C for 30 s, 55 °C for 30 s, and 72 °C for 30 s (35 cycles), and 72 °C for 10 min (1 cycle). ChIP primers for detailed sequences were shown in Supplementary Table S1.

### Data analysis

Statistical analysis was performed using the SPSS 17.0 statistical package (Chicago, IL, USA). Data were expressed as the mean ± SD. The Student’s *t*-test was used for comparison of two groups. Differences of multiple groups were compared using one-way analysis of variance (ANOVA). When ANOVA detects significant differences, the data were then compared using a Tukey’s test as post hoc test. Correlation coefficient (*r*) and *P*-values were calculated by Pearson’s correlation analysis. *P* < 0.05 was considered statistically significant.

## Results

### *PPARG2* is downregulated in PCA cell lines and tissues

**Fig. 1:**
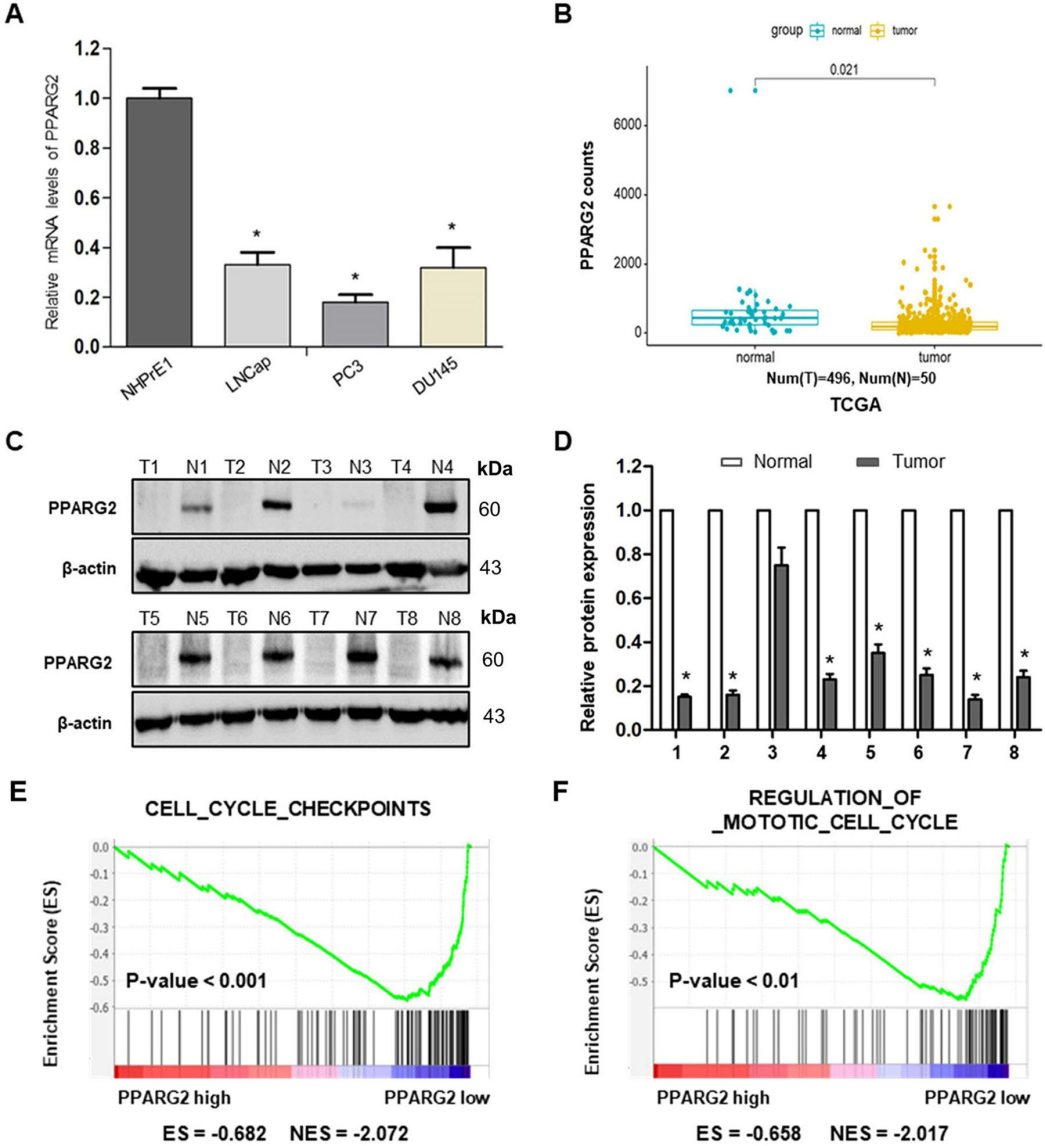
*PPARG2* is downregulated in PCA, negatively correlated with cell cycle-activated gene signatures. **A** Relative mRNA expression levels were analyzed in different PCA cell lines and a normal prostate epithelial progenitor cell line NHPrE1 by qRT-PCR. *P < 0.05 vs. normal group. **B** Expression of PPARG2 in 496 PCA tissues (tumor) compared with 50 normal tissues (normal) in the TCGA profile. *P* = 0.021. **C**, **D** Western blotting analyses of relative PPARG2 protein expression in eight PCA tissues (T) and eight prostate hyperplasia tissues (*N*). *P < 0.05 vs. normal group. **E**, **F** GSEA plot showing the PPARG2 expression level correlated negatively with cell cycle-activated gene signatures (CELL_CYCLE_CHECKPOINTS, REGULATION_OF_MITOTIC_CELL_CYCLE).

To determine the expression levels of *PPARG2* in PCA cell lines and tissues, we first detected the relative mRNA expression levels in different PCA cell lines (LNCap, PC3, and DU145) and a normal prostate epithelial progenitor cell line NHPrE1 by qRT-PCR. We found it was significantly downregulated in the three PCA cell lines compared with the normal cell line (Fig. 1A). Then, the data extracted from The Cancer Genome Atlas (TCGA) showed that PPARG2 was downregulated in PCA tissue samples (496 cases) compared with the normal tissues (50 cases) (Fig. 1B).

To examine the protein expression level of *PPARG2* in clinical PCA specimens, eight PCA tissues (T) and eight prostate hyperplasia tissues (N) were collected and analyzed. The results revealed lower PPARG2 protein expression was in the Tumor group than that in the Normal group (Fig. 1C, D). In addition, to obtain further insight into the function of *PPARG2*, the gene set enrichment analysis^26^ of TCGA profiles based on *PPARG2* single gene expression was performed. The results indicated that PPARG2 expression levels were correlated negatively with cell proliferation by affecting genes in cell cycle regulation (Fig. 1E, F). Taken together, the above results reveal obviously that *PPARG2* is downregulated in PCA.

### *PPARG2* suppresses cell migration, colony formation, invasion, and induces cell cycle arrest of PCA cells in vitro

**Fig. 2:**
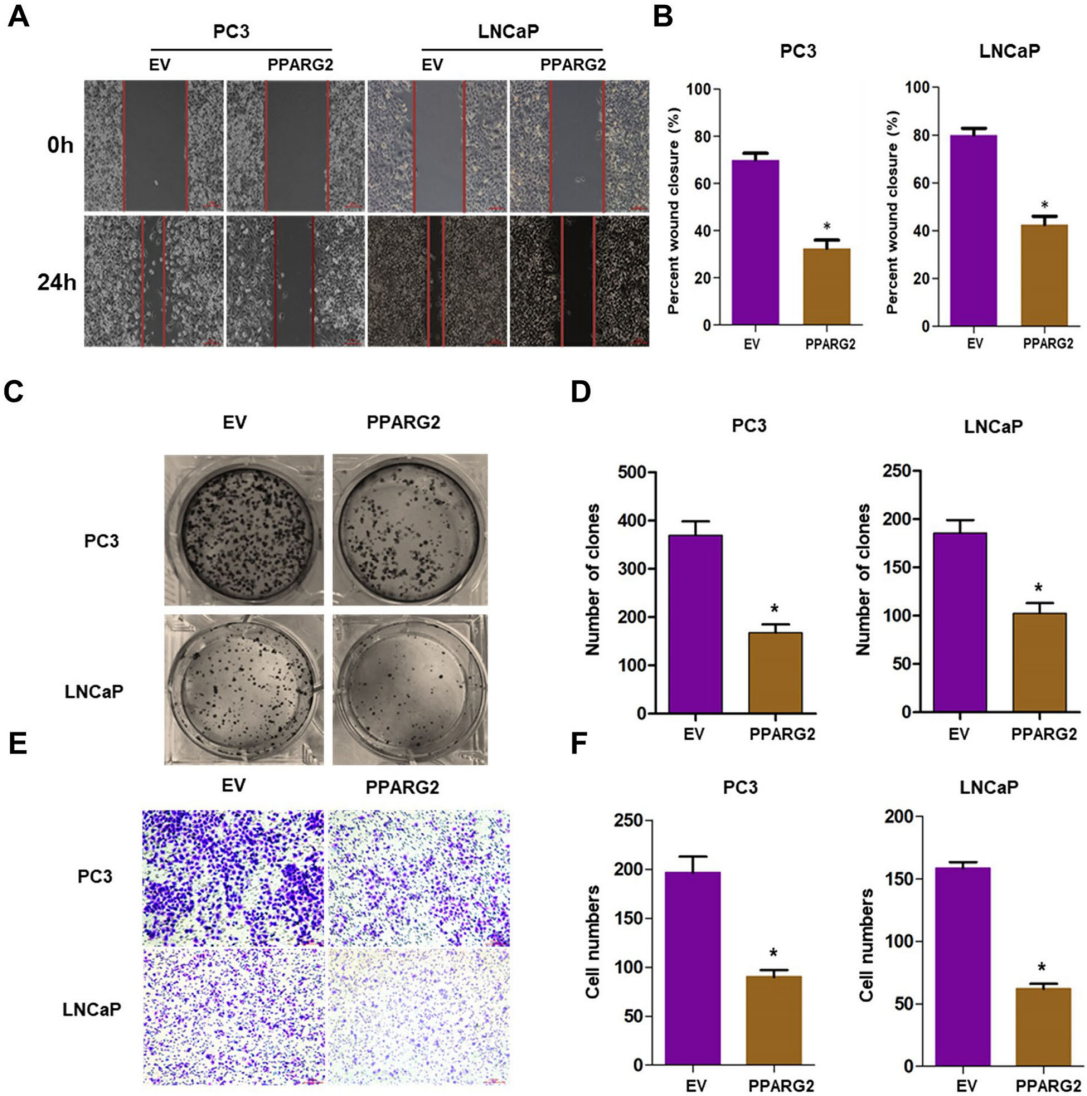
*PPARG2* suppresses cell migration, colony formation, and invasion in vitro. **A**, **B** Wound-healing assays were performed to detected the effects of *PPARG2* on the proliferation of the PCA cells. *P < 0.05 vs. EV group. **C**, **D** Colony formation assays were performed in EV and PPARG2-transfected PCA cells. *P < 0.05 vs. EV group. **E**, **F** Representative images of invasion PCA cells transfected with EV or PPARG2 overexpression lentivirus vectors by Transwell cell invasion tests.

To study the effects of *PPARG2* on the biological behaviors, PC3 and LNCaP cell lines were selected as research represents of PCA cells in the following studies. The *PPARG2*-overexpressing lentivirus vector-GV358 containing the human *PPARG2* wild-type full-length sequence (*PPARG2*) for gain-of-function and the lentivirus EV as a control were transfected respectively into the PCA cells. Results from wound-healing assays indicated that overexpression of *PPARG2* suppressed cell migration significantly in PC3 and LNCaP cell lines (Fig. 2A, B). Overexpression of *PPARG2* also significantly inhibited the colony numbers of PPARG2 group in the colony formation assay compared to those of the EV group (Fig. 2C, D). At the same time, Transwell cell invasion tests showed that the cells’ abilities of the *PPARG2* group to migrate and penetrate Matrigel was significantly reduced compared with those of the EV group (Fig. 2E, F). These results indicate that *PPARG2* may inhibit the proliferation and tumorigenicity of PCA cells.

**Fig. 3:**
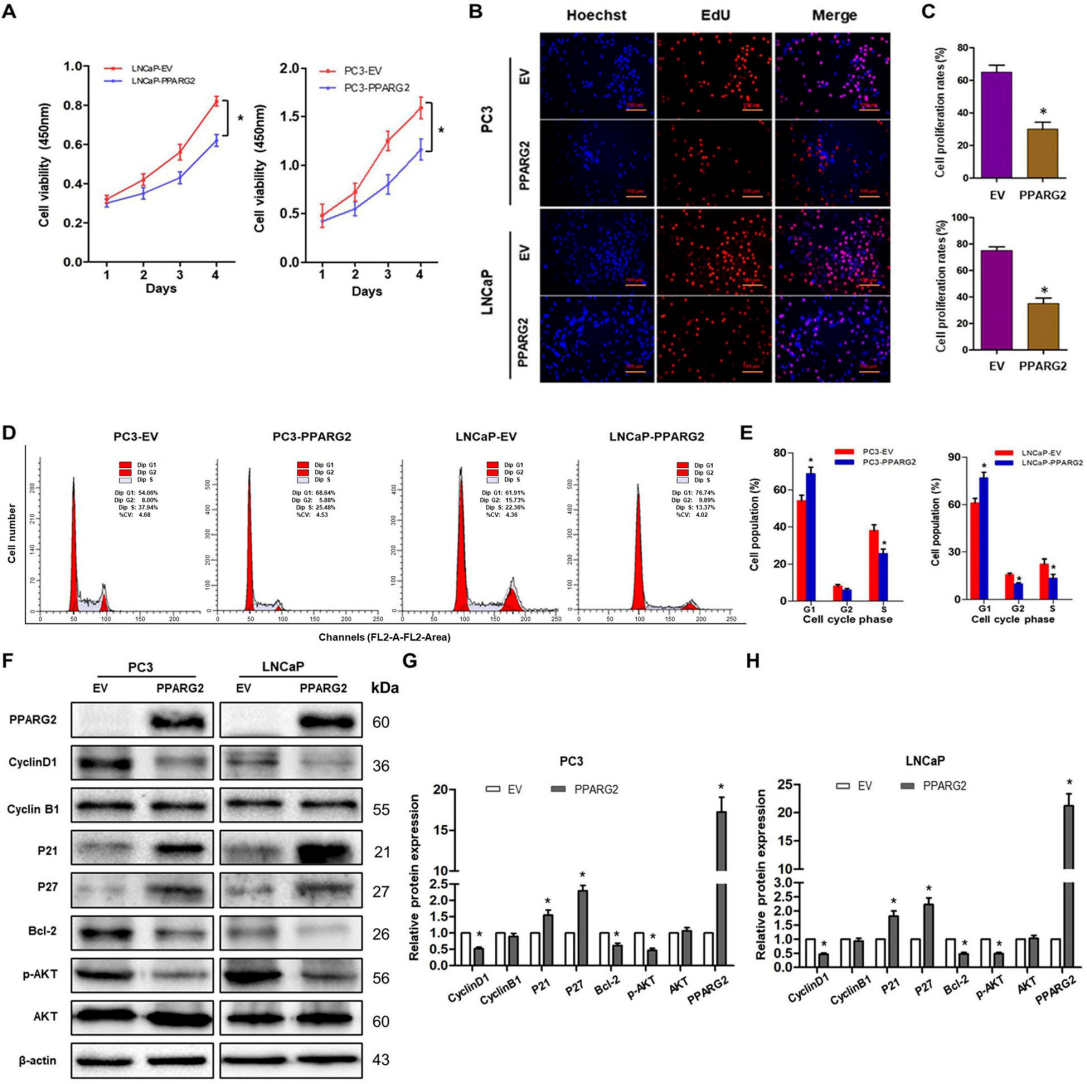
*PPARG2* suppresses cell proliferation and induces cell cycle arrest of PCA cells in vitro. **A** CCK-8 assay of the proliferation rates of recombinant PCA cell lines transfected with EV- and PPARG2-overexpression lentivirus vectors, respectively. *P < 0.01. **B**, **C** EDU assays were performed in EV and PPARG2-transfected PCA cells. *P < 0.05 vs. EV group. **D**, **E** Cell cycle profiles were examined by flow cytometry with propidium iodide (PI) staining; cell numbers were counted according to DNA content of G1, S, and G2 phases. *P < 0.05 vs. EV group. **F**–**H** Western blot analysis of cyclinD1, cyclinB1, p21^Cip1^ and p27^Kip1^, Bcl-2, p-AKT, and AKT protein expressions in indicated cells. *P < 0.05 vs. EV group.

To further evaluate the potential suppressing effects of *PPARG2* on cell proliferation, CCK-8 assay was performed in 1, 2, 3, and 4 days after Lv-PPARG2 and Lv-EV transfection. Compared with the EV group, a significant decrease of cell viability was detected in PC3 and LNCaP cells in the PPARG2 group (Fig. 3A). Then, EdU retention assays were performed to assess the inhibiting effect of PPARG2 on DNA replication. Following transfection with Lv-PPARG2, the percentage of EdU-positive cells was decreased significantly in PC3 and LNCaP cells compared to EV group (Fig. 3B, C).

Moreover, to identify the mechanism through which PPARG2 overexpression inhibits the proliferation of PCA cells, we checked the cell cycle distribution in PC3 and LNCaP cells transfected with Lv-PPARG2 or Lv-EV using flow cytometry (Fig. 3D, E). The results showed cell cycle of G1 arrest that the cell populations in the G1 phase of the cell cycle increased significantly in the PPARG2 group compared with EV-transfected controls of PC3 and LNCaP cells. However, the cell populations were reduced in the S stage of the cell cycle compared to the EV controls in the two cell lines. The results indicated that progression of G1-S cell cycle was inhibited by PPARG2 overexpression in PC3 and LNCaP cells. In addition, the protein expression levels of cyclinD1, cyclinB1, p21^Cip1^ and p27^Kip1^, Bcl-2, p-AKT, and AKT were also analyzed between the two groups in PC3 and LNCaP cells (Fig. 3F–H). Western blotting analysis revealed that cyclinD1, Bcl-2, and p-AKT were decreased significantly in PPARG2-transfected cells. Conversely, cell cycle inhibitors p21^Cip1^ and p27^Kip1^ were upregulated in PPARG2-transfected cells. Moreover, altering expression of PPARG2 in the two groups had no effect on the protein expression of AKT and cyclinB1.

### *PPARG2* inhibits tumor growth in vivo

**Fig. 4:**
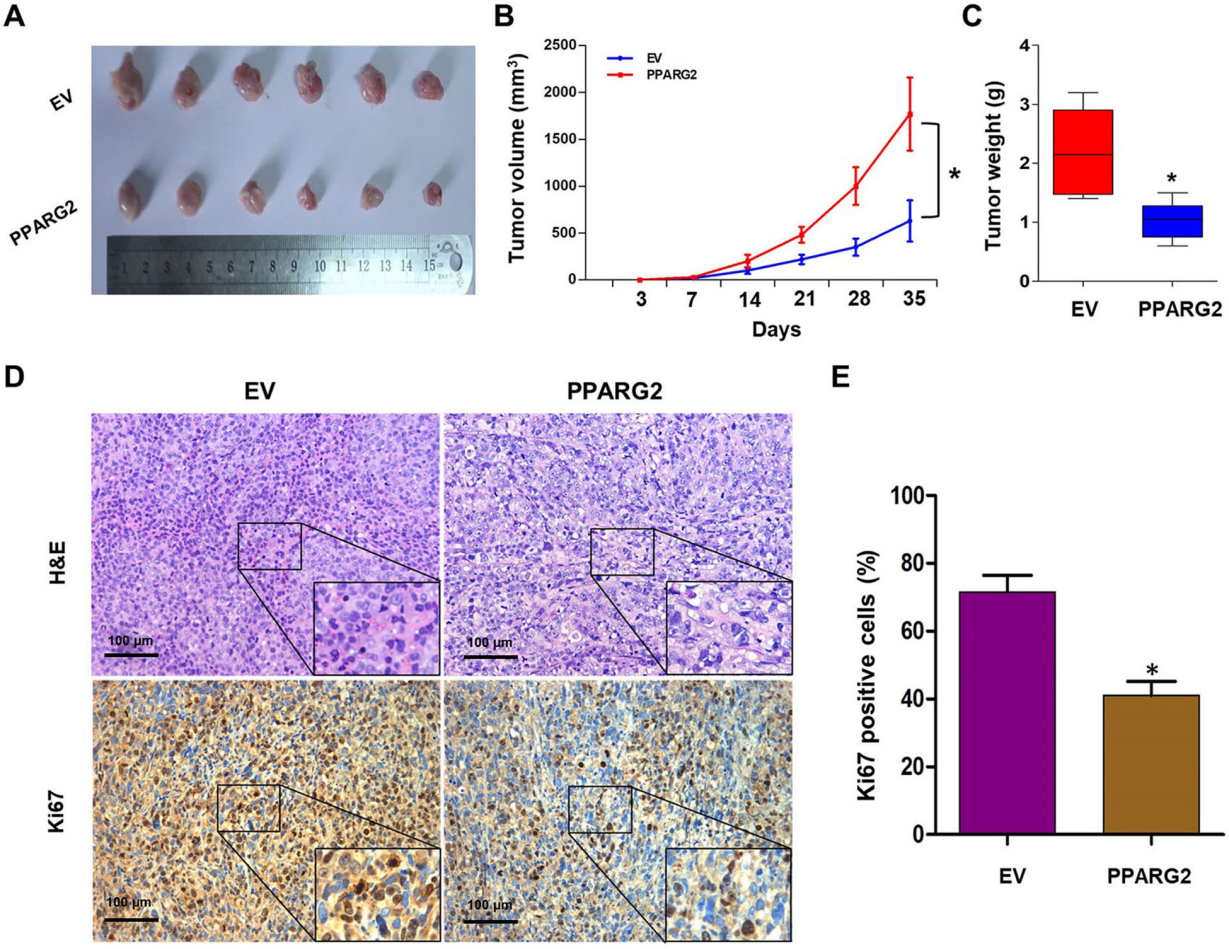
*PPARG2* inhibits PCA cell proliferation in vivo. **A**–**C** Representative xenograft tumors in nude mice that were injected PC3 cells subcutaneously with overexpression of transfected *PPARG2* or vector cells, respectively. Tumor volumes were calculated. Tumor weights were compared between EV and PPARG2 group. *P < 0.05 vs. EV group. **D**, **E** H&E staining and Ki67 expression level of subcutaneous tumors with IHC between EV and the PPARG2 group (×100). The representative images are from the same tumor samples. *P < 0.05 vs. EV group.

To further study the anticancer effects of *PPARG2* on PCA progression in vivo, xenograft models were established via injection subcutaneously of PC3 cells treated with *PPARG2* or EV into BALB/c nude mice. Compared with the EV group, the xenograft tumor volumes and weights in the PPARG2 group were all markedly decreased (Fig. 4A–C). Moreover, H&E and proliferating cell-associated antigen Ki67 staining were performed to study the proliferation level of the subcutaneous tumors. H&E-staining results showed the nuclei in both groups were large and deeply stained, whereas Ki67-staining results in tumor xenografts indicated that the Ki67-positive rate was decreased markedly in the PPARG2 group, suggesting that PPARG2 can inhibit tumorigenicity of PCA cells in vivo (Fig. 4D, E).

### *PPARG2*-mediated induction of *AKAP12* mRNA upregulation in vitro

**Fig. 5:**
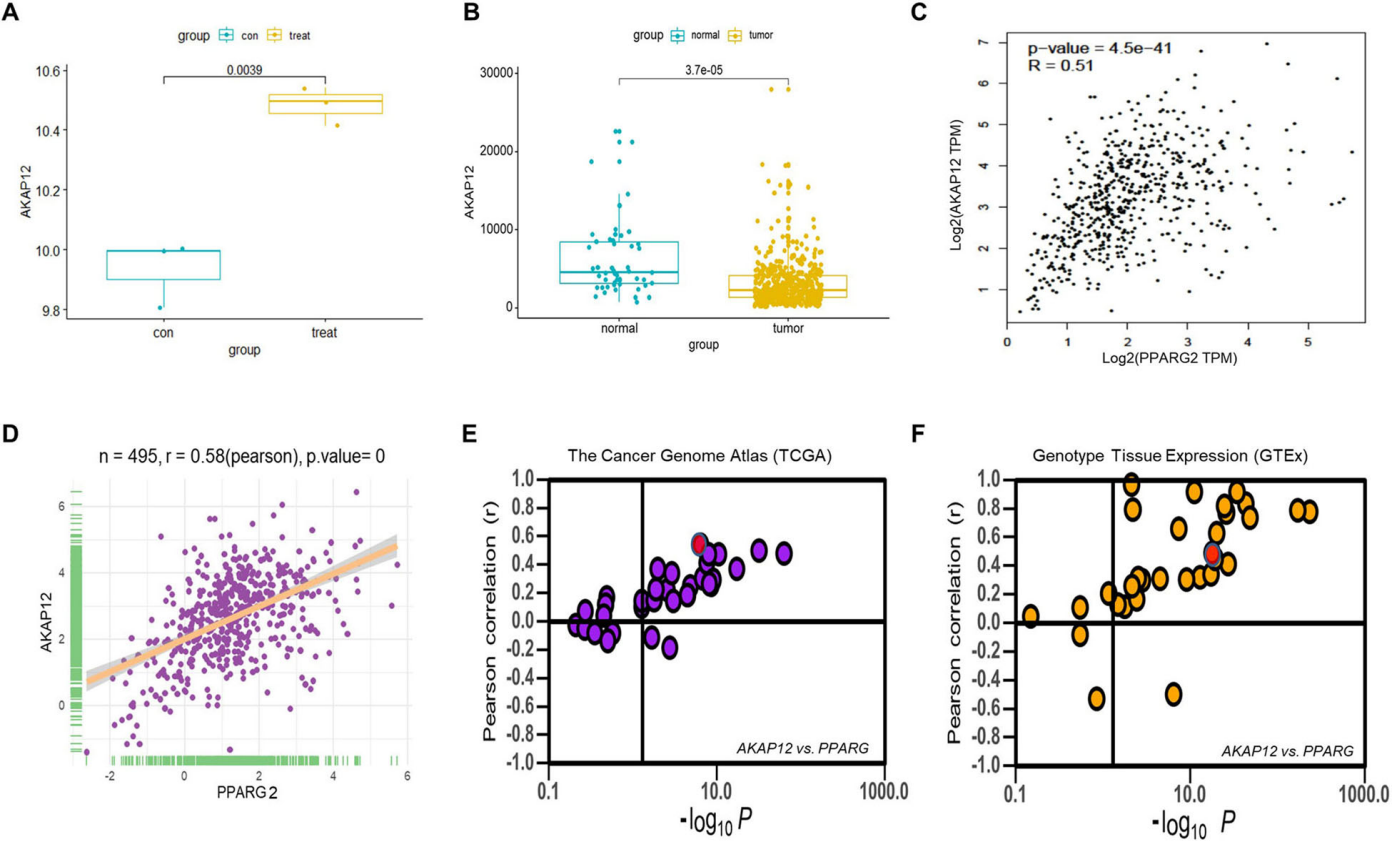
Positive correlation of *AKAP12* in mRNA expression with *PPARG2* gene in most cancer or normal tissues. **A** *AKAP12* mRNA expression extracted from the microarray data (EV (con) = 3, PPARG2 (treat) = 3, *P* = 0.0039). **B** *AKAP12* mRNA expression in PCA patients extracted from TCGA database (normal = 50, tumor = 496, *P* < 0.001). **C**, **D** Correlation of *AKAP12* with *PPARG2* in expression from GEPIA database (*r* = 0.51, *P* < 0.001) and TCGA database (*r* = 0.58, *P* = 0). **E**, **F** Correlation of *AKAP12* with *PPARG2* in expression in cancer samples from The Cancer Genome Atlas (TCGA database) and normal tissues from Genotype Tissue Expression (GTEx database), respectively. It is noteworthy that every dot represents one cancer type (E) in which the red dot represents PCA tissues (*r* = 0.58, *P* < 0.001) or one tissue type (F) in which the red dot represents prostate tissues (*r* = 0.57, *P* < 0.001).

To further explore the potential mechanisms of PPARG2-suppressing cell proliferation of PCA, gene expression microarray was performed using PC3 cells between overexpressing PPARG2 cDNA (PPARG2) and EV. The clustering heat map of differentially expressed genes between sample groups was shown in Supplementary Fig. S1A. Then, differentially expressed genes were screened to meet fold change > 1 and *P*-value < 0.05 between the two groups, of which 716 genes were upregulated and 822 genes were downregulated (Supplementary Fig. S1B). From the upregulated gene set, we screened the *AKAP12* gene (Fig. 5A) and the expression levels were significantly lower in PCA tissues than those in normal ones from the TCGA database (Fig. 5B). Moreover, correlation analysis from Gene Expression Profiling Interactive Analysis (GEPIA) (Fig. 5C) and TCGA (Fig. 5D) database indicated that the expressions between the two genes was positively correlated significantly. In addition, *PPARG2* also showed a positive correlation in mRNA expression with *AKAP12* in most cancer and normal tissues or cell lines (Fig. 5E, F). Then, functional analysis of GO enrichment and KEGG pathway were performed to investigate the functions and processes of the target gene set using the online software DAVID. The results revealed that GO enrichment involved in (i) biological process (BP) (Supplementary Fig. S2A), such as positive regulation of transcription, positive regulation of apoptotic process, and DNA methylation; (ii) involved in molecular function (Supplementary Fig. S2B), such as protein binding, TF binding, and poly(A) RNA binding; and (iii) involved in cellular component (Supplementary Fig. S2C), such as nucleoplasm, nucleus, and cytoplasm. The KEGG pathway analysis suggested that the differentially expressed genes of PC3-PPARG2 cells were involved in cell cycle, PI3K-Akt signaling pathway, and transcriptional misregulation in cancer, etc. (Supplementary Fig. S2D).

### Upregulated *PPARG2* induces demethylation of the *AKAP12* promoter region in vitro

From the above analysis results of BP, which is under GO classification, we found the target gene set were involved in DNA methylation. Then, expression levels of the three DNA methyltransferase (*DNMT1*, *DNMT3A*, and *DNMT3B*) were extracted from the microarray data. The results indicated that the expression levels of *DNMT3A* and *DNMT3B* were all downregulated markedly in the PPARG2 group compared with those of the EV group (Supplementary Fig. S3B, C), whereas *DNMT1* expression level showed a nonsignificant upregulation in the PPARG2-treated group (Supplementary Fig. S3A).

**Fig. 6:**
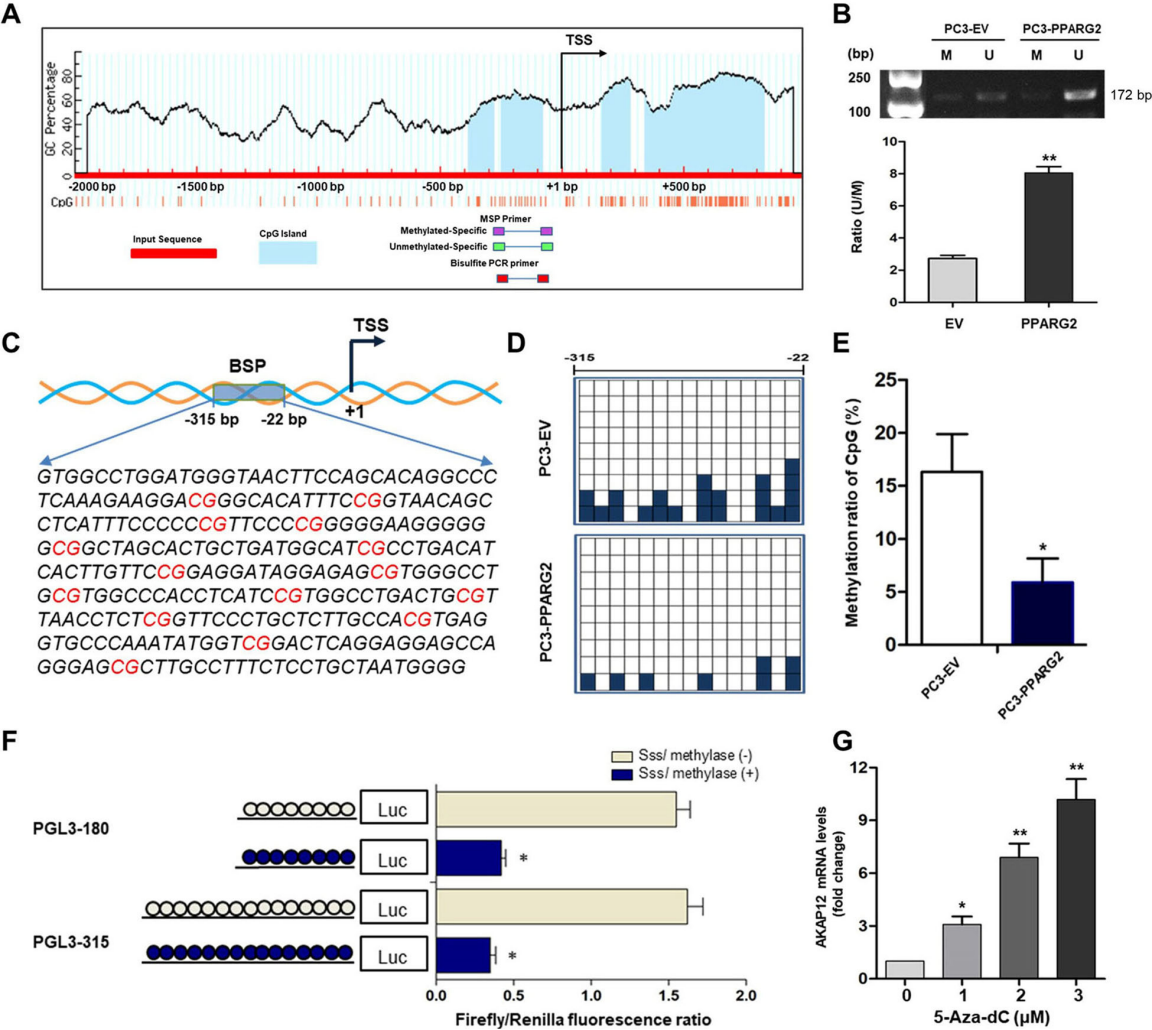
Upregulated *PPARG2* induces demethylation of the *AKAP12* promoter region in vitro. **A** Scheme for the location of the CpG islands near the TSS in *AKAP12* gene. The CpG sites were indicated by vertical red lines. The regions for MS-PCR and BSP were indicated. TSS, translation start site. **B** Representative results of methylation analysis by MS-PCR in PC3 cell line transfected with EV or PPARG2 plasmid. M, methylation; U, unmethylation. **P < 0.01 vs. EV group. **C** Schematic region from −315 to −22 bp for BSP including 15 CpG sites in the *AKAP12* gene promoter area. **D** BSP analysis of methylation status of the *AKAP12* gene promoter. It is noteworthy that each small square indicates one clone. A total of nine clones were subjected to bisulfite sequencing. The methylated clones of individual CpG sites are labeled in dark blue. **E** The methylated clone ratio at −315 to −22 bp region of CpG sites. *P < 0.05 vs. EV group. **F** Two luciferase constructs treated with *SssI* methylase in vitro. *P < 0.05 *vs. SssI* methylase (−) group. **G** RT-qPCR analysis of relative *AKAP12* mRNA expression in PC3 cell line treated with 5-Aza-dc at different concentrations. *P < 0.05, **P < 0.01 vs. group of 0 µM (5-Aza-dc).

To further investigate the intrinsic mechanism of *PPARG2*-induced upregulation of *AKAP12* and downregulation of *DNMT3A/3B*, we then conducted the following experiments to clarify it from the perspective of epigenetics. We first scanned the *AKAP12* promoter for potential regions of DNA methylation and found obvious CpG islands existed in the promoter region near the transcription start site (TSS) (Fig. 6A). Then, the CpG island demethylation statuses were detected between the PPARG2 and EV group by using MSP. The demethylation levels of the *AKAP12* promoter region were significantly increased in the PPARG2 group (Fig. 6B), which could be the result of downregulated DNMT3A/3B expression in the PPARG2 group. Moreover, to obtain further demethylation status details of specific CpG sites near the promoter region of *AKAP12* between the PPARG2 and EV group, a 294 bp length of PCR product (−315 to −22 bp) was analyzed following sodium bisulfite treatment using BSP method (Fig. 6C). The results revealed lower methylation frequencies in the PPARG2 group than those in the EV group (Fig. 6D, E).

In addition, to determine which CpG sites were responsible for the demethylation-related activation of the *AKAP12* gene under the condition of *PPARG2* upregulation in PCA cells, two *AKAP12* gene promoter regions (PGL3-180 and PGL3-315) were constructed and treated with *SssI* methylase in vitro before being transfected into the PC3 cell line (Fig. 6F). In comparison with the untreated promoter construct, the *SssI* methylase-treated construct showed a significant suppression of promoter activity. Although there were no significant differences in the promoter activity between the region of PGL3-180 and PGL3-315 with or without *SssI* methylase treatment, it indicated that the promoter region at −180 bp may play an important role in regulating *AKAP12* gene transcription. Finally, relative *AKAP12* mRNA expression levels were detected after PC3 cells were treated with different concentrations (0, 1, 2, and 3 μM) of DNMT inhibitor 5-Aza-2′-deoxycytidine (5-Aza-dc). The detection results showed a 5-Aza-dc dose-dependent significant upregulation of *AKAP12* mRNA expression levels (Fig. 6G), which indicated that methylation statuses of the related region in *AKAP12* promoter were involved in regulation of its expression.

### Upregulation of miR-200b-3p regulates expression of the downstream target genes *DNMT3A/3B* in PPARG2-overexpressed PCA cells

**Fig. 7:**
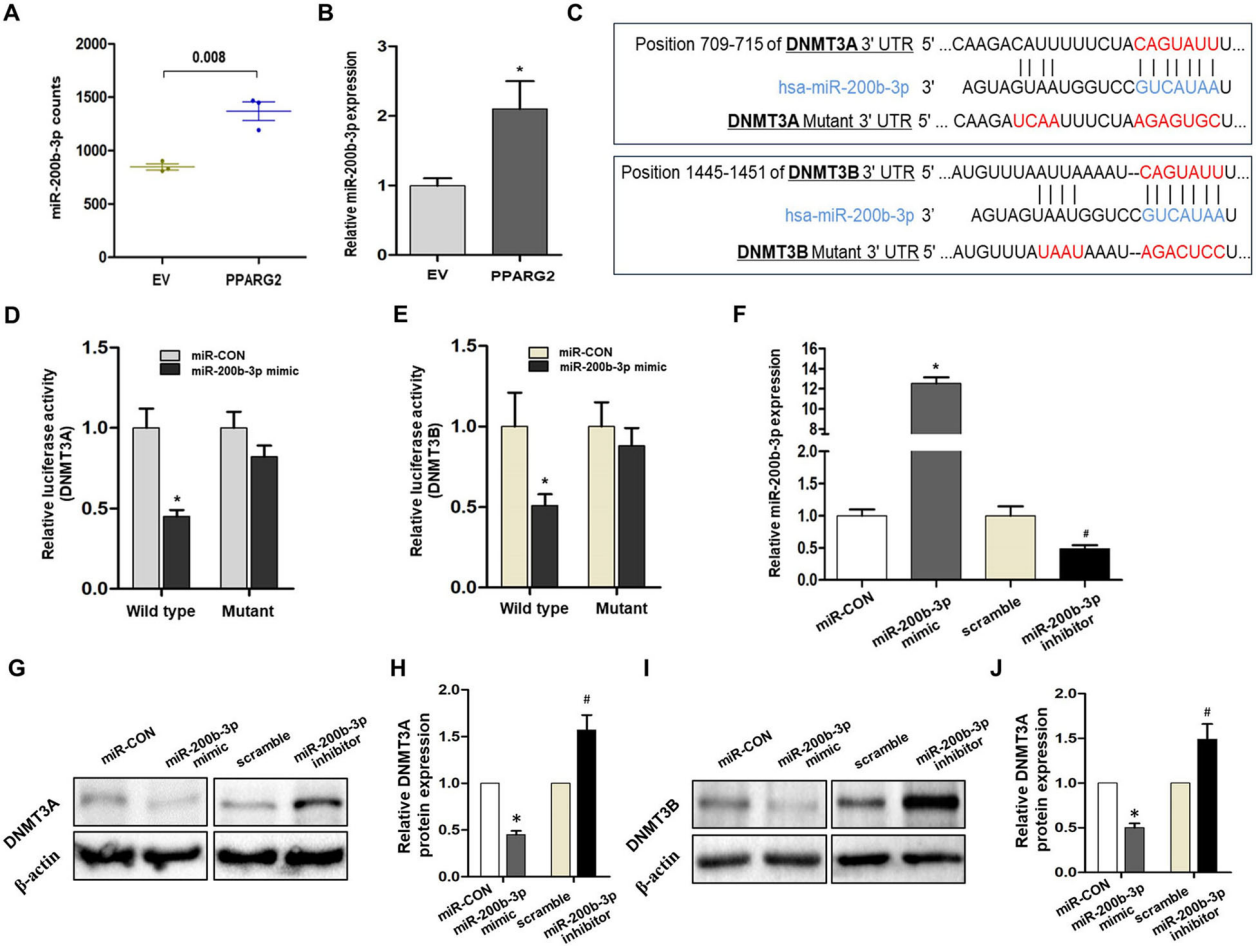
Experimental verification of the relationship between DNMT3A/3B and miR-200b-3p. **A** miR-200b-3p expression extracted from miRNA-seq results (*n* = 3, *P* = 0.008). **B** Relative miR-200b-3p expression in vitro when overexpression of *PPARG2*. *P < 0.05 vs. EV group. **C** Putative miR-200b-3p-binding 3′-UTR sequence of *DNMT3A/3B* mRNA. Mutation was generated on the *DNMT3A/3B* mRNA 3′-UTR sequence in the complementary site for the seed region of miR-200b-3p. The wild-type or mutant miR-200b-3p-binding *DNMT3A/3B* mRNA 3′-UTR sequence was cloned into pmiR luciferase reporter. **D** The wild-type (DNMT3A/3′-UTR-WT) and mutant (DNMT3A/3′-UTR-Mut) pmiR luciferase reporter were co-transfected into PC3 cells with miR-CON and miR-200b-3p. *P < 0.05 vs. miR-CON group. **E** The wild-type (DNMT3B/3′-UTR-WT) and mutant (DNMT3B/3′-UTR-Mut) pmiR luciferase reporter were co-transfected into PC3 cells with miR-CON and miR-200b-3p. *P < 0.05 vs. miR-CON group. **F** Relative miR-200b-3p expression in vitro transfected with miR-200b-3p mimic or miR-200b-3p inhibitor by RT-qPCR. U6 was used as an internal control. *P < 0.05 vs. miR-CON group, ^#^*P* < 0.05 vs. scramble group. **G**, **H** Analysis of DNMT3A protein expression by western blotting when transfected with miR-CON, miR-200b-3p mimic, scramble, or miR-200b-3p inhibitor into PC3 cells. β-Actin was used as an internal control. *P < 0.05 vs. miR-CON group, ^#^*P* < 0.05 vs. scramble group. **I**, **J** Analysis of DNMT3B protein expression by western blotting when transfected with miR-CON, miR-200b-3p mimic, scramble, or miR-200b-3p inhibitor into PC3 cells. β-Actin was used as an internal control. *P < 0.05 vs. miR-CON group, ^#^*P* < 0.05 vs. scramble group.

To further explore the mechanism of upregulated *PPARG2* causing downregulation of *DNMT3A/3B*, one of the possibilities we thought was that miRNAs may be involved in the regulatory function of the genes. Then, miRNA-seq was performed between PPARG2 and EV group of the PC3 cell line. The clustering heat map of differentially expressed miRNAs between sample groups were shown in Supplementary Fig. S4A. Differentially expressed miRNAs were screened, and 18 miRNAs were upregulated and 44 miRNAs were downregulated (Supplementary Fig. S4B). From the upregulated 18 miRNAs, we identified miR-200b-3p as the study target, which was upregulated significantly in the PPARG2 group compared with the EV group extracted from miRNA-seq results (Fig. 7A), and it has been then confirmed by experimental verification (Fig. 7B).

The next step, the regulatory relationship between miR-200b-3p and *DNMT3A/3B*, was confirmed via experimental verification. Bioinformatics revealed that *DNMT3A/3B* 3′-region contained one conserved target site of miR-200b-3p, respectively (Fig. 7C). To evaluate this prediction, the wild-type 3′-UTR sequence of *DNMT3A/3B* (wild type) or its mutant sequence (Mut) (Fig. 7D, E) was subcloned into the pmiR luciferase reporter and then co-transfected with miR-CON or miR-200b-3p mimic into 293T cells. The results indicated that the relative luciferase activity of the pmiR wild type was decreased significantly by 45.0% (Fig. 7D) and 51.3% (Fig. 7E), respectively, when miR-200b-3p mimic was co-transfected into the cells. However, no differences of relative luciferase activity of pmiR-Mut showed when co-transfected with miR-CON or miR-200b-3p mimic into PC3 cells.

Moreover, the qPCR-detected results verified that relative miR-200b-3p expression levels showed significant upregulation or downregulation in miR-200b-3p mimic or miR-200b-3p inhibitor-treated PC3 cells (Fig. 7F). Finally, we examined DNMT3A/3B expression in PC3 cells by western blotting. As expected, miR-200b-3p mimic triggered a significant suppressing effect on DNMT3A/3B protein expression. However, the protein expression levels of DNMT3A/3B were regained in miR-200b-3p inhibitor-treated PC3 cells (Fig. 7G–J). The results further confirmed the regulatory effect of miR-200b-3p on DNMT3A/3B mRNA.

### Enhanced binding of *PPARG2* to *AKAP12* promoter region in PPARG2-overexpressed PC3 cells

**Fig. 8:**
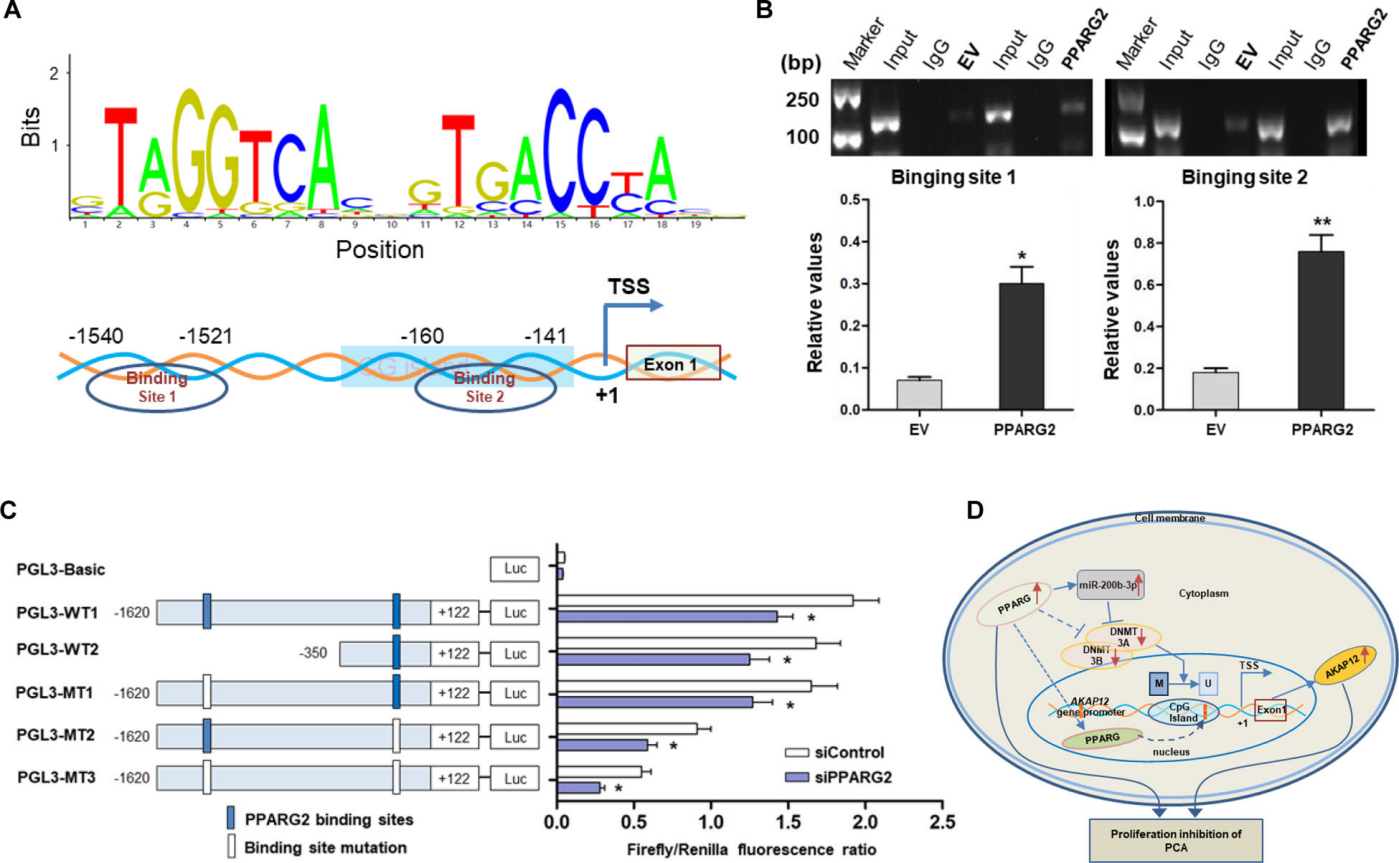
Enhanced binding of *PPARG2* to *AKAP12* promoter region due to DNA demethylation when *PPARG2* is upregulated. **A** Scheme for binding sequence and sites of *PPARG2* binding to the transcription factor-binding site near the *AKAP12* gene promoter region. TSS, transcription start site. **B** ChIP assay revealing the direct binding of PPARG2 to the *AKAP12* gene promoter in PC3 cells between the EV and PPARG2 group. The enriched DNA fragments within the *AKAP12* gene promoter using IgG and an anti-PPARG2 antibody were amplified by PCR. Total input was used as a positive control. *P < 0.05, **P < 0.01 vs. EV group. **C** Serially truncated and mutated *AKAP12* gene promoter constructs were co-transfected with siPPARG2 or siControl into PC3 cells, and the relative luciferase activities were determined. *P < 0.05 vs. siControl group. **D** Proposed graphic model for PPARG2-AKAP12 axis-mediated epigenetic regulation mechanism of proliferation, migration, and invasion in PCA cells.

To further explore the mechanism by which *PPARG2* induces the upregulation of *AKAP12*, we thought of *PPARG2*, which, as a kind of TF, may be involved in binding to the promoter region of *AKAP12*. We predicted the potential TF-binding site near the *AKAP12* promoter region using the online software JASPAR (http://jaspar.genereg.net/) and found two potential *PPARG2*-binding sites (site 1, −1540 to −1521 bp and site 2, −160 to −141 bp) (Fig. 8A). Then ChIP assay was performed to verify the prediction. The results revealed that *PPARG2* could bind to the two binding sites. Meanwhile, enhanced binding of *PPARG2* can be found in *PPARG2*-overexpressed PC3 cells (Fig. 8B).

Moreover, to further verify the efficiency of the binding sites from ChIP assay results, PGL3 plasmids containing serially truncated and mutated *AKAP12* gene promoter were constructed and co-transfected with siControl or siPPARG2 into 293T cells, to determine the most effective functional binging site that caused *PPARG2*-regulated *AKAP12* gene promoter activation (Fig. 8C). Luciferase reporter activity detection results showed a significant reduction of promoter activity in the cells that co-transfected with siPPARG2 of both PGL3-WT and PGL3-MT groups. At the same time, the reduced promoter activity caused by sequence truncation was not very obvious. From the luciferase results of serially mutated *AKAP12* gene promoters (pGL3-MT1, pGL3-MT2,and pGL3-MT3), a more significant reduction in AKAP12 gene promoter activity was found in pGL3-MT2 (site 1, −160 to −141 bp) or pGL3-MT3 (two sites mutation simultaneously). The results suggested that the *PPARG2*-binding site 2 may play a more important role in *AKAP12* transcriptional activation.

## Discussion

Peroxisome proliferator-activated receptors (PPARs) are involved in many diseases such as cancer^27,28^, diabetes^29^, and inflammation^30^. Studies have revealed that PPARG acts as a tumor suppressor and plays an important role in tumorigenesis^31,32^. Additional studies showed PPARG as an oncogene in the development of tumors^33,34^. Regarding the dual role of PPARG gene played in the occurrence and development of tumors, we believe it may be related to multiple factors involved in tumor types and tumor progression in a specific environment. In this study, our research target PPARG2, one of the PPARG protein isoforms, was found to be downregulated in PCA and inhibited cell migration, colony formation, invasion, and induced cell cycle arrest of PCA cells in vitro. Moreover, *PPARG2* overexpression modulates the activation of the Akt signaling pathway, as well as inhibit tumor growth in vivo. This is our first report to elaborate the functional significance of *PPARG2* expression in human PCA and experiment results indicated that *PPARG2* functioned as a tumor suppressor and inhibits PCA malignant progression. Thus, *PPARG2* holds great prospects as a new therapeutic target for PCA.

As TFs, most of members of PPARs can bind to the specific sites of the target gene promoter region to regulate their expression^35^. Therefore, for the next step for mechanism exploration, we screened differentially expressed genes via gene expression microarray from the PPARG2 and EV groups in vitro first. We found that *AKAP12* mRNA was upregulated, mediated by PPARG2 overexpression. Reports have revealed that *AKAP12* is a protein kinase C substrate and a potential tumor suppressor in many types of cancers including hepatocellular carcinoma (HCC)^36^, colorectal cancer^37^, and breast cancers^38^. Moreover, interestingly, GO enrichment results indicated *PPARG2* overexpression involved in DNA methylation of BP. Accordingly, we then associated *AKAP12* gene with DNA methylation. We found that obvious CpG islands existed in its promoter region near the TSS and confirmed that upregulated *PPARG2* induced demethylation of the *AKAP12* promoter region via MSP and BSP experiments. Moreover, relative AKAP12 mRNA expression levels increased in a 5-Aza-dc dose-dependent concentration, which indicated that methylation statuses of *AKAP12* promoter were involved in regulation of its expression. The above results indicated PPARG2-AKAP12 axis-mediated epigenetic regulatory network may play a role in PCA.

Enzymes that catalyze CpG methylation in DNA sequence include DNA methyltransferase 1 (DNMT1), DNMT3A, and DNMT3B. These DNA methyltransferases are essential for mammalian tissue development and homeostasis, and also related to human developmental disorders and cancer, which supports that DNA methylation plays a key role in the specification and maintenance of cell fate^39,40^. In this study, we found that expression levels of *DNMT3A* and *DNMT3B* were all downregulated markedly in the PPARG2 group compared with those in EV the group. This data coincided with the upregulation of *AKAP12* due to its promoter demethylation.

The next step, we explored the potential mechanism of *DNMT3A/3B* downregulation induced by *PPARG2* overexpression in PCA. It may be two ways: a direct regulation or an indirect effect through a mediator. Regarding the possible indirect influence mechanism, miRNAs could be suitable mediators, which belong to noncoding RNAs family. They mainly cause mRNA translational repression or degradation by targeting to the 3′-UTR of mRNAs^41^. Thus, we screened out miR-200b-3p using miRNA-seq and the *DNMT3A/3B* 3′-UTR region contains conserved target sites of miR-200b-3p. Studies have shown that miR-200b-3p was downregulated in androgen-independent PCA^42^, HCC^43^, and glioma^44^. Our experiments confirmed the regulatory relationship between miR-200b-3p and *DNMT3A/3B*. The data indicated that miR-200b-3p participated indirectly in PPARG2-AKAP12 axis-mediated epigenetic regulatory network in PCA.

In addition, the ChIP results demonstrated the enhanced binding of PPARG2 to *AKAP12* gene promoter in *PPARG2*-overexpressed PC3 cells and the sequential deletion and mutation analyses revealed that binding site 2 (−160 to −141 bp) may play a more important role in the *AKAP12* gene promoter activity. Coincidentally, the binding site 2 is exactly where CG island is located. It is obvious that increased affinity of *PPARG2* to the specific binding site is due to the CG island demethylation. The data revealed that DNA demethylation enhanced the binding of *PPARG2* to the *AKAP12* gene promoter and participated in regulation of the *AKAP12* gene expression.

In summary, the present work elucidates a PPARG2-AKAP12 axis-mediated epigenetic regulatory network in PCA (Fig. 8D). *PPARG2* acts as a tumor suppressor in suppressing malignancy of PCA cells in vitro and in vivo. As a transcriptional factor, *PPARG2* overexpression induces the increased expression level of miR-200b-3p, which targeted 3′-UTR of the downstream targets *DNMT3A/3B*, facilitates interaction with demethylated *AKAP12* gene promoter and suppresses cell proliferation in PCA via AKT signaling pathway. Of course, we recognize that upregulated *PPARG2* potentially regulate a cluster of miRNAs, while one miRNA can target lots of genes, and involves in cross-talk pathways in the complex and elaborate epigenetic regulatory network in PCA. Therefore, we need to make more efforts to better explore the sophisticated mechanisms of *PPARG2* regulation in the progression of PCA. In brief, our findings provided the first evidence for a novel PPARG2-AKAP12 axis-mediated epigenetic regulatory network. The study identified a molecular mechanism involving an epigenetic modification that could be possibly targeted as an antitumoral strategy against prostate PCA.

## Supplementary information

Supplemental figure and table legends

Fig. S1. Clustering and screening of differentially expressed genes between EV and PPARG2 groups.

Fig. S2. Functional analysis of GO enrichment and KEGG pathway of target gene set.

Fig. S3. Expression levels of DNA methyltransferase extracted from the microarray data.

Fig. S4. Clustering and screening of differentially expressed miRNAs between EV and PPARG2 groups.

Supplementary Table S1: Primers sequences used in this study
