# Supplementary material for: Upregulated PPARG2 facilitates interaction with demethylated AKAP12 gene promoter and suppresses proliferation in prostate cancer
Source: Cell Death Dis. 2021 May 22;12(6):528. doi: 10.1038/s41419-021-03820-7 (PMC8141057; doi:10.1038/s41419-021-03820-7)
Supplement: Supplementary file 1 — Supplemental figure and table legends [file 41419_2021_3820_MOESM1_ESM.docx]

**Fig. S1 Clustering and screening of differentially expressed genes between** **EV and PPARG2 groups. A** Clustering heat map of differentially expressed genes between sample groups (EV vs PPARG2). **B** Volcano map of differentially expressed genes between EV and PPARG2 groups.

**Fig. S2 Functional analysis of GO enrichment and KEGG pathway of target gene set. A** Biological Process (BP) analysis under GO classification. **B** Molecular Function (MF) analysis under GO classification. **C** Cellular Component (CC) analysis under GO classification. **D** Functional analysis of KEGG pathway of the target gene set.

**Fig. S3 Expression levels of DNA methyltransferase extracted from the microarray data.** **A** *DNMT1* gene expression extracted from the microarray data (EV (con) =3, PPARG2 (treat) =3, *P* = 0.023). **B** *DNMT3A* gene expression extracted from the microarray data (EV (con) =3, PPARG2 (treat) =3, *P* = 0.0059). **C** *DNMT3B* gene expression extracted from the microarray data (EV (con) =3, PPARG2 (treat) =3, *P* = 0.0091).

**Fig. S4 Clustering and screening of differentially expressed miRNAs between EV and PPARG2 groups. A** Clustering heat map of differentially expressed miRNAs between sample groups (EV vs PPARG2). **B** Volcano map of differentially expressed miRNAs between EV and PPARG2 groups.

**Table S1: Primers sequences used in this study**
