# Supplementary figures and images for: Upregulated PPARG2 facilitates interaction with demethylated AKAP12 gene promoter and suppresses proliferation in prostate cancer
Source: Cell Death Dis. 2021 May 22;12(6):528. doi: 10.1038/s41419-021-03820-7 (PMC8141057; doi:10.1038/s41419-021-03820-7)

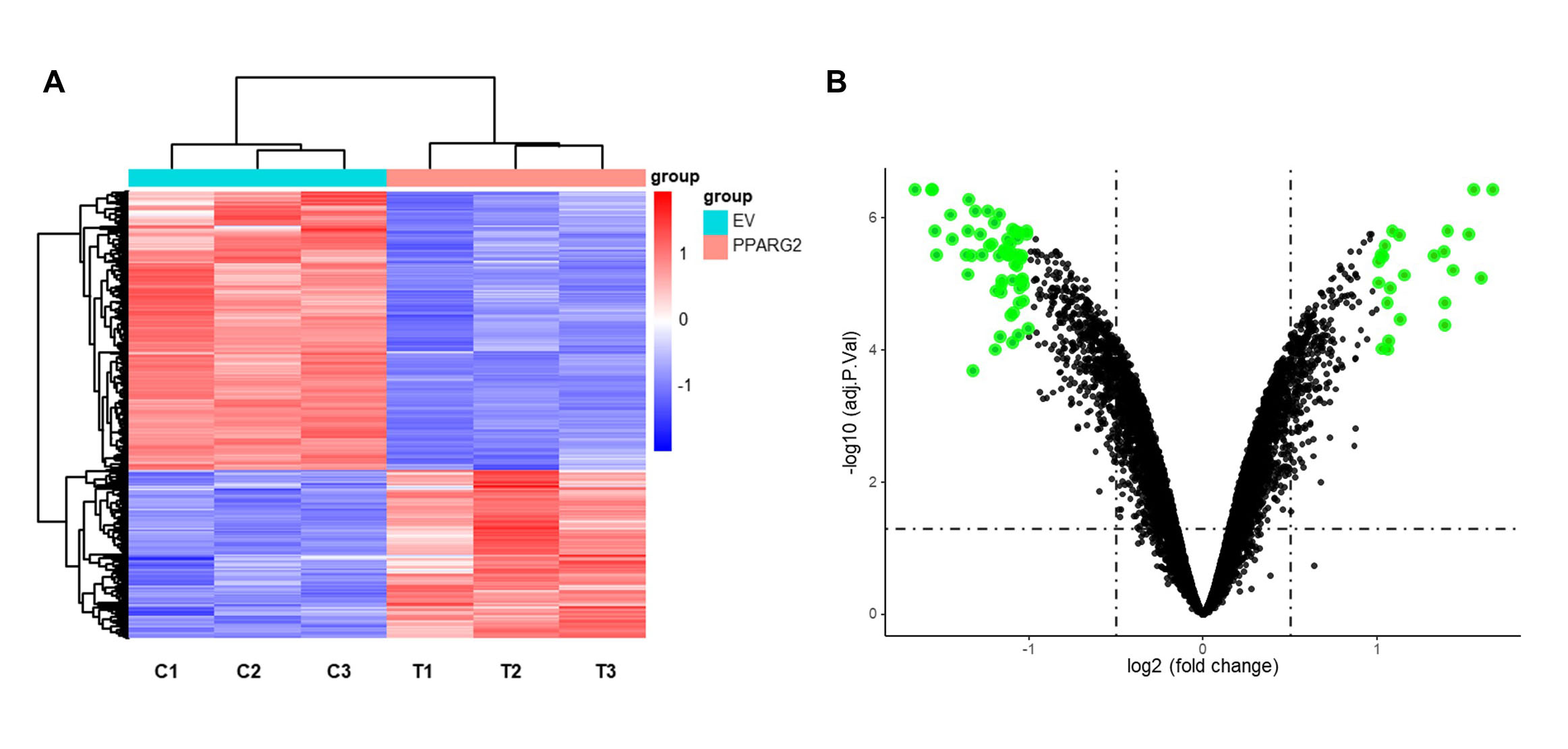

Supplement: Supplementary file 2 — Fig. S1. Clustering and screening of differentially expressed genes between EV and PPARG2 groups. [file 41419_2021_3820_MOESM2_ESM.jpg]

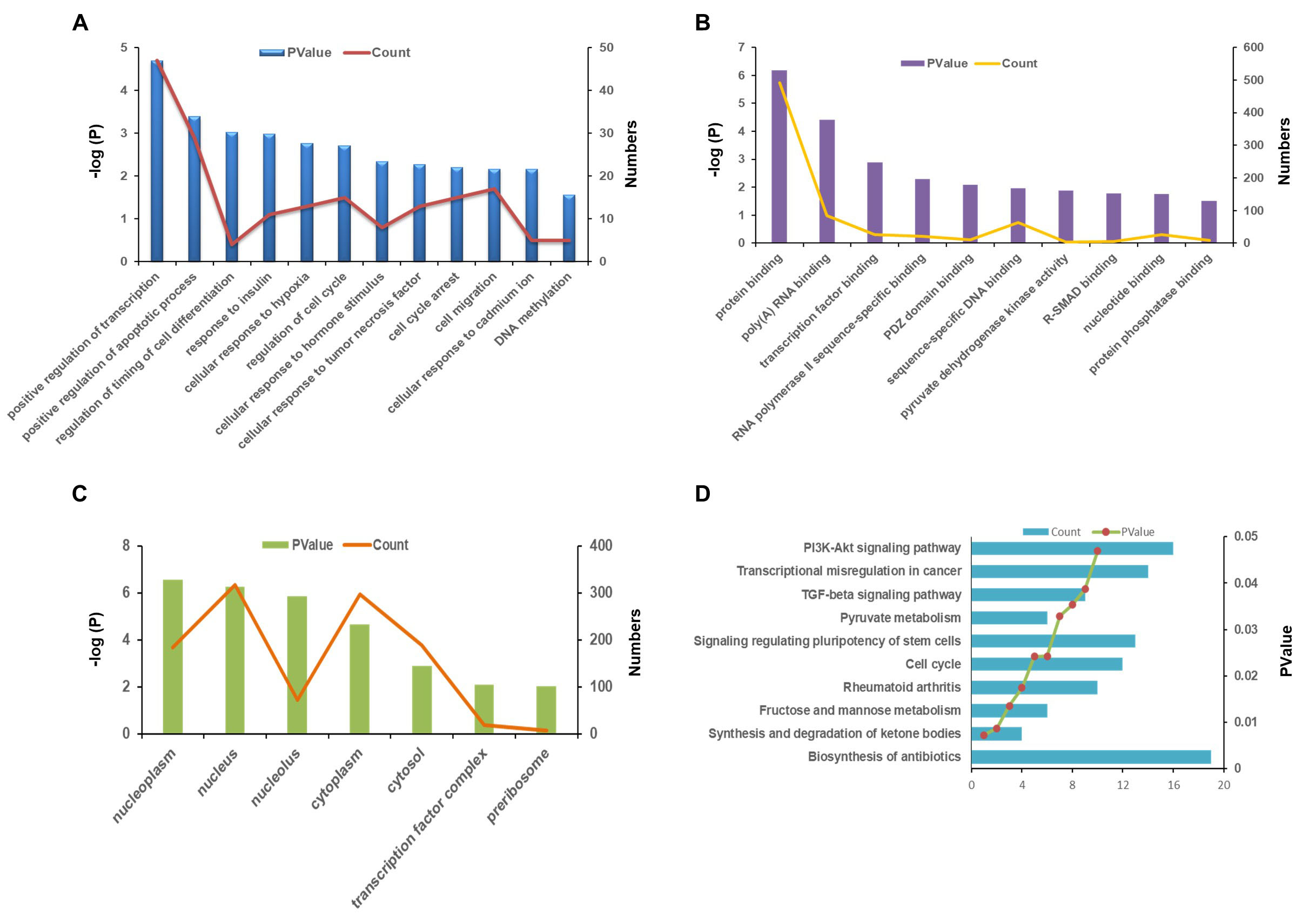

Supplement: Supplementary file 3 — Fig. S2. Functional analysis of GO enrichment and KEGG pathway of target gene set. [file 41419_2021_3820_MOESM3_ESM.jpg]

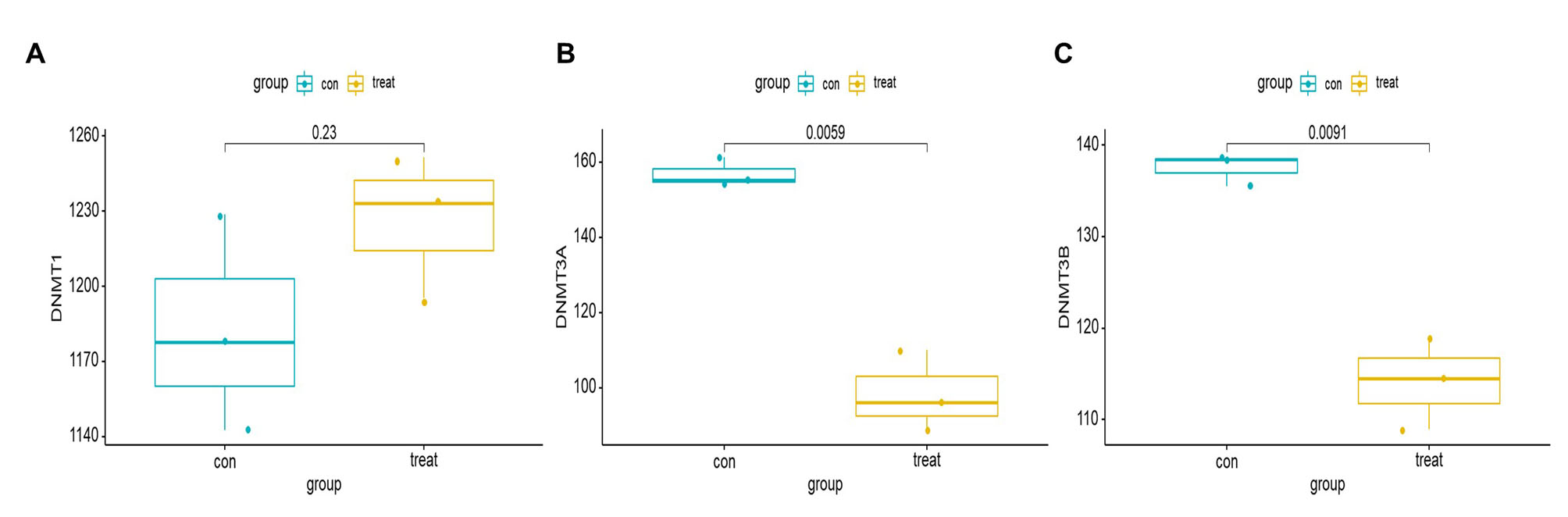

Supplement: Supplementary file 4 — Fig. S3. Expression levels of DNA methyltransferase extracted from the microarray data. [file 41419_2021_3820_MOESM4_ESM.jpg]

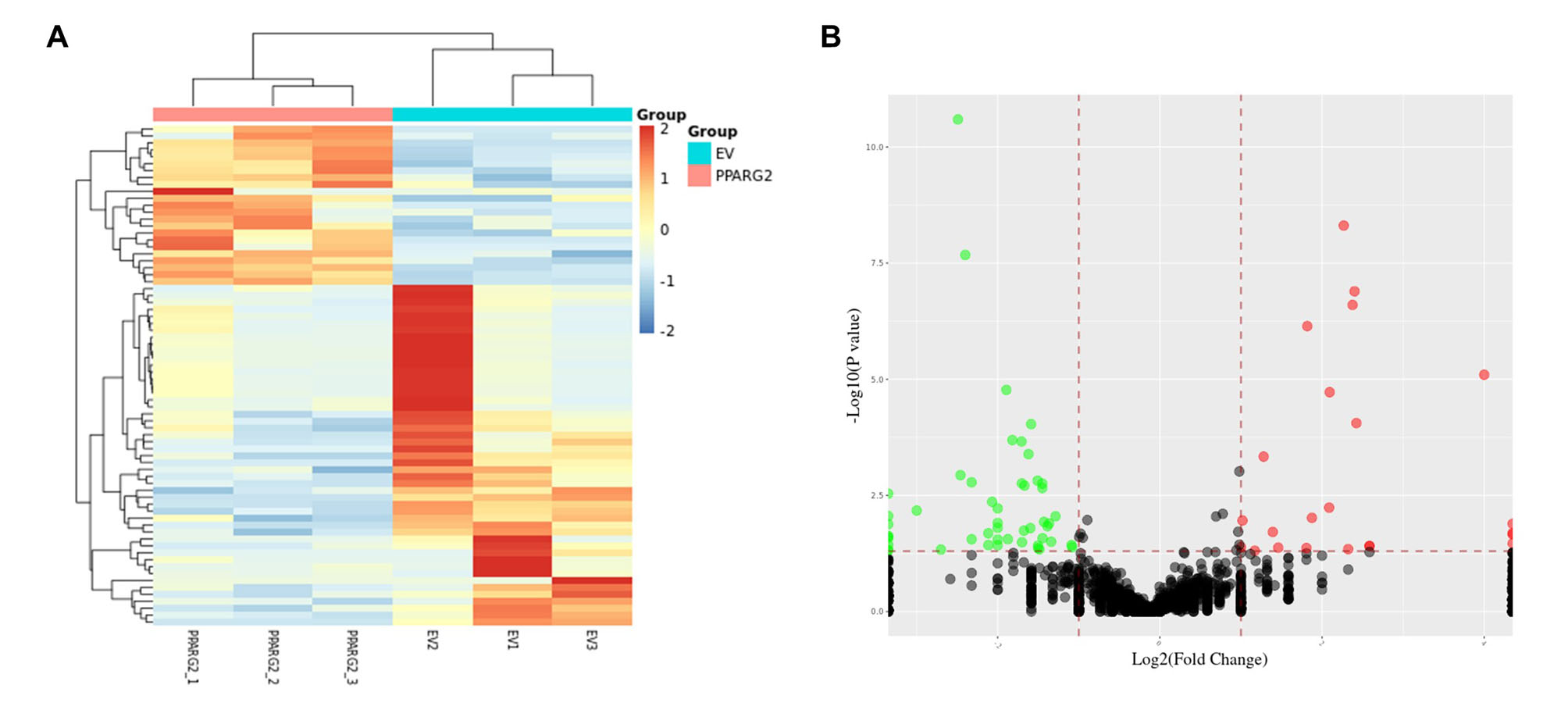

Supplement: Supplementary file 5 — Fig. S4. Clustering and screening of differentially expressed miRNAs between EV and PPARG2 groups. [file 41419_2021_3820_MOESM5_ESM.jpg]
