# Supplementary material for: Upregulated PPARG2 facilitates interaction with demethylated AKAP12 gene promoter and suppresses proliferation in prostate cancer
Source: Cell Death Dis. 2021 May 22;12(6):528. doi: 10.1038/s41419-021-03820-7 (PMC8141057; doi:10.1038/s41419-021-03820-7)
Supplement: Supplementary file 6 — Supplementary Table S1: Primers sequences used in this study [file 41419_2021_3820_MOESM6_ESM.docx]

**Supplementary Table S1: Primers sequences used in this study**

| **Primer name** | **sequences（5’→3’）** |
| --- | --- |
| **Gene and miRNA** |  |
| PPARG2 | F: AGAAAGCGATTCCTTCACTGAT  R: AGAATGGCATCTCTGTGTCAAC |
| AKAP12 | F: GTCTCCTTCATTCGCAGGCT  R: CATGGCTCCTCCGCACTTCTC |
| GAPDH | F: TGACTTCAACAGCGACACCCA  R: CACCCTGTTGCTGTAGCCAAA |
| miR-200b-3p | F: CGCCATCTGCCTCGACTAC  R: CTGGCTCTCCACCATCTTCTGC |
| U6 | F: CTCGCTTCGGCAGCACA  R: AACGCTTCACGAATTTGCGT |
| **siRNA** |  |
| si-PPARG2 | F: GCUCCACACUAUGAAGACAUUTT  R: AAUGUCUUCAUAGUGUGGAGCTT |
| si-Control | F: GUACCUGACUAGUCGCAGAAG  R: GUACCUGACUAGUCGCAGAAG |
| **MSP and BSP** |  |
| *AKAP12*-MSP-M | F1: AGTATAGGTTTTTAAAGAAGGACGG  R1: AAACAAAAAACCGAAAAATTAACG |
| *AKAP12*-MSP-U | F1: AGTATAGGTTTTTAAAGAAGGATGG  R1: AACAAAAAACCAAAAAATTAACACA |
| *AKAP12* -BSP-F： | F1: GTGGTTTGGATGGGTAATTTT  R1: CCCCATTAACAAAAAAAAAACA |
| **ChIP** |  |
| P1 | F: ATTGAAAGGGAAGGGGAGT  R: TTACCATTGGCAGGAGGG |
| P2 | F: CATCGCCTGACATCACTTG  R: CCATTAGCAGGAGAAAGGC |
